# Comparing the effects of augmented virtual reality treadmill training versus conventional treadmill training in patients with stage II-III Parkinson’s disease: the VIRTREAD-PD randomized controlled trial protocol

**DOI:** 10.3389/fneur.2024.1338609

**Published:** 2024-01-24

**Authors:** Gemma Lombardi, Marco Baccini, Alice Gualerzi, Silvia Pancani, Silvia Campagnini, Stefano Doronzio, Diego Longo, Alessandro Maselli, Giulio Cherubini, Michele Piazzini, Tommaso Ciapetti, Cristina Polito, Samuele Pinna, Chiara De Santis, Marzia Bedoni, Claudio Macchi, Silvia Ramat, Francesca Cecchi

**Affiliations:** ^1^Department of Neurosciences, Psychology, Drug Research and Child Health (NEUROFARBA), University of Florence, Florence, Italy; ^2^IRCCS Fondazione Don Carlo Gnocchi Onlus, Florence, Italy; ^3^IRCCS Fondazione Don Carlo Gnocchi Onlus, Milan, Italy; ^4^Department of Experimental and Clinical Medicine, University of Florence, Florence, Italy; ^5^Department of Technical-Health Professions, Rehabilitation, and Prevention, Campostaggia Hospital, Poggibonsi (SI), USL Toscana Sudest, Italy; ^6^Parkinson Unit, Department of NeuroMuscular-Skeletal and Sensorial Organs, AOU Careggi, Florence, Italy

**Keywords:** Parkinson’s disease, gait, balance, falls, rehabilitation, treadmill, biomarkers, Extracellular Vesicles

## Abstract

**Background:**

Intensive treadmill training (TT) has been documented to improve gait parameters and functional independence in Parkinson’s Disease (PD), but the optimal intervention protocol and the criteria for tailoring the intervention to patients’ performances are lacking. TT may be integrated with augmented virtual reality (AVR), however, evidence of the effectiveness of this combined treatment is still limited. Moreover, prognostic biomarkers of rehabilitation, potentially useful to customize the treatment, are currently missing. The primary aim of this study is to compare the effects on gait performances of TT + AVR versus TT alone in II-III stage PD patients with gait disturbance. Secondary aims are to assess the effects on balance, gait parameters and other motor and non-motor symptoms, and patient’s satisfaction and adherence to the treatment. As an exploratory aim, the study attempts to identify biomarkers of neuroplasticity detecting changes in Neurofilament Light Chain concentration T0-T1 and to identify prognostic biomarkers associated to blood-derived Extracellular Vesicles.

**Methods:**

Single-center, randomized controlled single-blind trial comparing TT + AVR vs. TT in II-III stage PD patients with gait disturbances. Assessment will be performed at baseline (T0), end of training (T1), 3 (T2) and 6 months (T3, phone interview) from T1. The primary outcome is difference in gait performance assessed with the Tinetti Performance-Oriented Mobility Assessment gait scale at T1. Secondary outcomes are differences in gait performance at T2, in balance and spatial–temporal gait parameters at T1 and T2, patients’ satisfaction and adherence. Changes in falls, functional mobility, functional autonomy, cognition, mood, and quality of life will be also assessed at different timepoints. The G*Power software was used to estimate a sample size of 20 subjects per group (power 0.95, *α* < 0.05), raised to 24 per group to compensate for potential drop-outs. Both interventions will be customized and progressive, based on the participant’s performance, according to a predefined protocol.

**Conclusion:**

This study will provide data on the possible superiority of AVR-associated TT over conventional TT in improving gait and other motor and non-motor symptoms in persons with PD and gait disturbances. Results of the exploratory analysis could add information in the field of biomarker research in PD rehabilitation.

## 1 Introduction

Parkinson’s Disease (PD) is a complex neurodegenerative disorder that affects from 1 to 2% of people over 65 years of age (1), characterized by multiple motor and non-motor symptoms (2). Among motor disturbances, gait disturbance is the key symptom of the disease, which can affect step length, walking speed, trunk oscillations, and arms’ pendular movements; freezing and festination phenomena are possible. Gait disturbance is often accompanied by alterations in posture and balance, and can be associated with falls, with a decisive impact on the autonomy and quality of life (QoL) of the patient and caregiver (3). The control of motor symptoms related to PD is mainly carried out with pharmacological therapy; in recent years, deep brain stimulation has also acquired an important role in the advanced phases of the disease (4). However, these interventions have some limitations, especially in the presence of balance and gait disturbances, and the literature highlights the importance of integrating rehabilitation into the care of patients with PD (5–7). Intensive walking training in PD has evidence of effectiveness, in particular in stride speed and length, on activities of daily living (8, 9) and in reducing falls (10). Among the rehabilitation interventions for improving walking in PD, conventional treadmill training (TT) has resulted to be more effective compared to no-TT rehabilitation in improving gait speed and stride length with moderate and low-quality evidence, respectively (11), highlighting the need to confirm these results with higher evidence and to establish an optimal intervention protocol including treatment personalization (12).

Regarding non-motor symptoms, cognitive impairment may associate or even precede motor symptoms in PD and can worsen over time from mild cognitive impairment to full-blown dementia during the disease course. Drugs give limited benefits to this disorder, making it essential to explore the effects of non-pharmacological approaches (13). Targeted cognitive training presents some evidence of effectiveness in patients with initial impairment, but a recent review suggests that TT produces even greater cognitive effects (14). Virtual reality (VR) is used in the motor and cognitive training of patients with various neurological diseases, including PD. VR refers to a computer-generated three-dimensional digital environment that can be explored and interacted with by a person. Augmented reality or augmented virtual reality (AVR) is the union between VR and real life. The person who experiences it can interact with virtual content in the real world. Computer-generated content is superimposed on reality through visual stimuli such as objects and films, tactile and olfactory sensations, and sounds. AVR is proposed as an interactive approach, that aims to amplify the effects of motor training, both directly and indirectly, increasing the patient’s motivation and satisfaction, promoting greater adherence and participation to treatment, and training also cognitive aspects of fall risk (14). For these reasons, AVR is considered an emerging and promising therapeutic tool in neurorehabilitation (15). In this regard, positive results emerged from the clinical trial of Gulcan et al. (16) in which AVR-VR TT has been demonstrated to be effective in PD on most of spatial–temporal gait parameters compared to conventional physical therapy.

Moreover, results from recent reviews dedicated to comparing the benefit of VR rehabilitation, including TT, versus conventional one showed that VR appears to be promising in motor outcomes such as gait and mobility (17–19), obtaining the most replicated and convincing result on balance (18–21); less evidence has been reported on functional autonomy (19) and QoL (18).

The Motek C-Mill is a recently developed treadmill for assessing and training gait and balance. By integrating AVR, audible and visual cues, and force platform technology, C-Mill enables obstacle avoidance training, dual-tasking applications, realistic VR environments, and a variety of balance challenges to promote balance strategies and gait adaptation and prepare patients to regain walking in daily life, using a safe and controlled environment. The integration with a force platform allows the acquisition of the trajectory of the center of pressure, automatically adapting the complexity of the tasks based on patient performance in real-time and returning feedback to the patient and the therapist to monitor progress and performance. The device was developed to treat gait disorders of various origins; in particular, the C-Mill has been used to study obstacle avoidance difficulties in PD (22, 23), but its effectiveness in the rehabilitation of these patients needs to be confirmed. Focusing on the comparison between conventional TT versus TT+AVR treatment effect on PD motor symptoms, current results are in favor of the TT+AVR for the gain obtained in incident falls in the long period (6 months) and in gait adaptability, a key target for reducing fall risk (22). These data come from 2 studies conducted on older at risk of falling, including cases affected by PD and results have been obtained using a quantitative method of gait assessment, the GaitRite mat (23, 24), preliminary data offering promising results on a possible benefit of interventions that combine TT and AVR in PD (25).

The integration of VR on TT in PD rehabilitation is an extremely current topic in PD rehabilitation research. Both interventions separately showed positive results on gait parameters (23–26), in particular stride speed and length and also balance, however, larger studies with a randomized controlled design are necessary to define recommendations for the use of VR and TT, isolated and in combination, in PD rehabilitation, to be applied according also to the patient characteristics.

Indeed, since the effect of rehabilitation may be different across motor subtypes (27) and disease severity (8), treatment personalization holds promise in PD rehabilitation. However, a timely optimization of personalized rehabilitation treatment for PD patients is nowadays at least in part limited by the lack of measurable biomarkers indicative of neuroplasticity phenomena and predictive of the response to the rehabilitation treatment. In this regard, Neurofilament Light Chain (NfL) has been recognized as a non-specific marker indicative of axonal damage in different neurological diseases (28–30), whereas it is not fully understood whether and how its concentration changes after rehabilitation treatment. In late phase after stroke the adaptive synaptic plasticity is supposed to be the main mechanism of NfL release, suggesting that this biomarker could also be useful to track the positive effects following rehabilitation (29). Recent literature suggests that neuroplasticity phenomena can be investigated with other innovative approaches, spanning from quantification of single molecules with highly sensitive technologies to more complex biomarkers like miRNome profile and circulating Extracellular Vesicles (cEVs) characterization. The study of the cellular processes taking place in the central nervous system in patients with PD before, during, and after rehabilitation might provide useful tools to correlate the severity of the damage and the peculiarities of the patient with the effectiveness of treatment. cEVs are natural nanoparticles defined as membrane-bound nanovesicles, released by body cells under physiological and pathological conditions as vehicles for bioactive molecules useful for intercellular communication. It has recently been demonstrated that biological fluids are rich in such vesicles, in particular vesicles originating from all the organs of our body, including the central nervous system, circulate in the blood (31). cEVs may change in concentration, dimension and biochemical content as a consequence of pathological processes and possibly after rehabilitation. In patients with PD, vesicles appear to play a role in the transport of molecules involved in the pathogenetic mechanisms of the disease itself, such as for example in the transport of the alpha-synuclein protein at the cerebral and peripheral level (32). The presence of cEVs involved in the pathogenesis and evolution of the disease and carriers of potential diagnostic markers has been demonstrated in peripheral blood (33). However, the size, heterogeneity, and large number of molecules present within and on cEVs have limited their use for diagnostic purposes. Raman spectroscopy is proposed as a useful method for the rapid and exhaustive biochemical characterization of circulating exosomes without the use of staining and labeling procedures. It has already been demonstrated how the Raman signature of cEVs can be correlated with some clinical scales commonly used for PD patient profiling, suggesting their potential use for patient stratification (34).

In summary, the evidence of the potential superiority of TT + AVR versus TT to improve gait in PD is still preliminary, and few studies include both clinical and instrumental gait measures, as it is instead recommended (3); further, an optimal TT(+AVR) protocol, customized to patients’ performances has not been established; finally, potential biomarkers of neuroplasticity and prognostic biomarkers are still under study.

Taking into account these considerations, the primary objective of this study is to compare the effects on gait performances of an integrated rehabilitation intervention before and after intensive rehabilitation with TT endowed of AVR, versus TT alone in patients with PD in II-III Hoehn and Yahr stage (35) with gait disturbances. The secondary objectives are to assess the effects of the interventions on balance performances, kinematic gait parameters and other motor and non motor symptoms immediately after treatment and at a 3-months follow up, and to assess the patient’s satisfaction and adherence to the treatment. The exploratory objective is to investigate potential serum biomarkers of neuroplasticity and prognosis.

## 2 Methods and analysis

Consensus meetings were held with movement disorders experts (Azienda Ospedaliero Universitaria Careggi, Parkinson Unit, Florence), guided by a Neurologist (S.R.) with more than 20 years expertise in PD, rehabilitation experts (Department of experimental and clinical medicine, University of Florence, IRCCS Fondazione Don Carlo Gnocchi of Florence), and biologists of the Laboratory of Nanomedicine and Clinical Biophotonics (LABION, IRCCS Fondazione Don Carlo Gnocchi of Milan), guided by M.B. with more than 10 years of expertise in biomarkers research, to develop an extensive and feasible assessment protocol with the following requirements: (1) to identify variables that can be easily and reliably collected in an inpatient rehabilitation setting, using internationally recommended and validated tools, adopting, when available, Italian versions, (2) to gain objective measures of clinical and functional variables through instrumental assessment (3) to monitor changes in biological markers potentially indicative of neuroplasticity or predictive of rehabilitation outcomes.

In more detail, the clinical assessment protocol has been developed in collaboration with the Parkinson Unit by Neurologists, Neuropsychologists, Physiatrists and Physiotherapists with long-lasting clinical and research expertise in neurological rehabilitation; the rehabilitation intervention has been developed in collaboration with the Parkinson Unit and will be carried out by Physiotherapists with at least 5 years of expertise in the rehabilitation of PD patients; all assessments will be performed by researchers with similar clinical experience and background in neurorehabilitation research, blind to treatment assignment.

### 2.1 Study design

This is a single-center, no-profit, parallel arm randomized controlled single-blind trial with an active comparator where the experimental intervention is represented by an TT+AVR and the comparator by a conventional TT.

Randomization will be performed by an external Researcher (not involved in the study design or procedures): a list of 0–1 will be generated using Phyton. Allocation will be concealed. Medical staff will propose consecutively the study for all eligible patients referring to the Parkinson Unit and the IRCCS Don Gnocchi Florence, assigning, in case of enrollment, an identification number to the participant.

Outcome raters will be blinded to the treatment assignment, including clinicians assessing the eligibility of patients and the raters (Neurologist, Physiatrist, Psychologist, Physiotherapist, Laboratory staff). Blindness will be ensured by the modality and timing of assessments at T0, T1, and T2 that will be conducted at times other than treatment and by raters not involved in the training. Blinding will be removed if requested by the participant at the end of the study and in case of undesirable effects.

The promoter of the study is the Department of Clinical and Experimental Medicine, University of Florence, Italy; the study will be performed at “Struttura Organizzativa Dipartimentale (SOD) di Riabilitazione Generale-Istituto di Ricovero e Cura a Carattere Scientifico (IRCCS) Fondazione Don Carlo Gnocchi, Florence.”

Two collaborators for external services will take part in the study:

- The Movement Analysis Laboratory at IRCCS Don Carlo Gnocchi Florence, dedicated to instrumental data analysis (postural sway and gait analysis).
- The Laboratory of Nanomedicine and Clinical Biophotonics (LABION) at IRCCS Fondazione Don Carlo Gnocchi, in Milan, dedicated to biological data analysis.

The study received Ethical Committee approval from the local Institutional Review Board (Comitato Etico Regione Toscana – Area Vasta Centro) on the 5th of July 2022 (Approval Number: 20915_spe).

The registration on ClinicalTrials.gov has been completed and the study has been reviewed obtaining the identification number “NCT05902065.”

### 2.2 Sample size estimation

The sample size was estimated based on the primary outcome of the study “the evaluation of the difference in gait assessed by the Tinetti Performance-Oriented Mobility Assessment gait (POMA-G) scale (36) between the TT+AVR and the TT group at T1.” Data for the estimation were obtained from a previous study aiming to evaluate the effects of non-conventional TT (partial weight-supported TT) on patients with PD (37) using the POMA-G scale. Mean values observed in the POMA-G score after 4 weeks of treatment were 10.7 ± 1.1 and 11.7 ± 0.5, for the conventional gait training and partial weight-supported TT group, respectively, leading to a high effect size *d* = 1.17. Based on those data and assuming a statistical power of 95% and an *α* = 0.05, the resulting sample was 20 subjects per arm. Dropouts are expected to be around 20%. To compensate for possible dropouts, the enrolment of a further 8 patients has been deemed appropriate, reaching an estimate of 24 subjects per group. Thus, 48 patients will be enrolled starting from the date of Ethical Committee approval, within July 2024. The sample size has been estimated using G*Power software (38).

### 2.3 Inclusion and exclusion criteria

Individuals with idiopathic PD consecutively referring for counseling and outpatient rehabilitation management will be included, if they fulfill the following inclusion criteria:

- diagnosis of PD according to the POSTUMA diagnostic criteria (39), in II-III Hoehn and Yahr stage (35), in stable drug therapy for at least 1 month
- age more than 18
- gait disturbance
- ability to walk for at least 5 min without assistance
- willing to participate in the study and ability to understand and sign informed consent

Individuals will be excluded in the presence of:

- other pathologies able to interfere with motor skills as symptomatic arthritis involving hip/knee/ankle, stroke outcomes or severe polyneuropathy
- cognitive impairment potentially interfering with rehabilitation procedures, estimated as a corrected score of less than 18.58 at the Montreal Cognitive Assessment (MoCA) (40)
- hallucinations and other psychiatric disorders not controlled by drug therapy, as in case of alcohol or drug abuse
- uncompensated visual/auditory deficit that limits the enjoyment of the cues provided by the AVR
- communication deficit from any cause that impairs understanding of the task and the objectives of the intervention
- medical conditions hindering the effect of the training as severe orthostatic hypotension and severe cardiovascular diseases.

Patients undergoing other experimental protocols will also be excluded, whereas patients who regularly engage in physical activity or sport will not be excluded.

Pharmacological treatment is required to be stable until T1. During the study, patients will be excluded in case of insufficient adherence to the intervention according to the study procedures. Missing up to 5 sessions is allowed, and lost sessions will be possibly made up at the end of treatment. Participants who miss more than 5 sessions will be considered dropouts.

### 2.4 Assessment and timeline of the study

**Figure 1:**
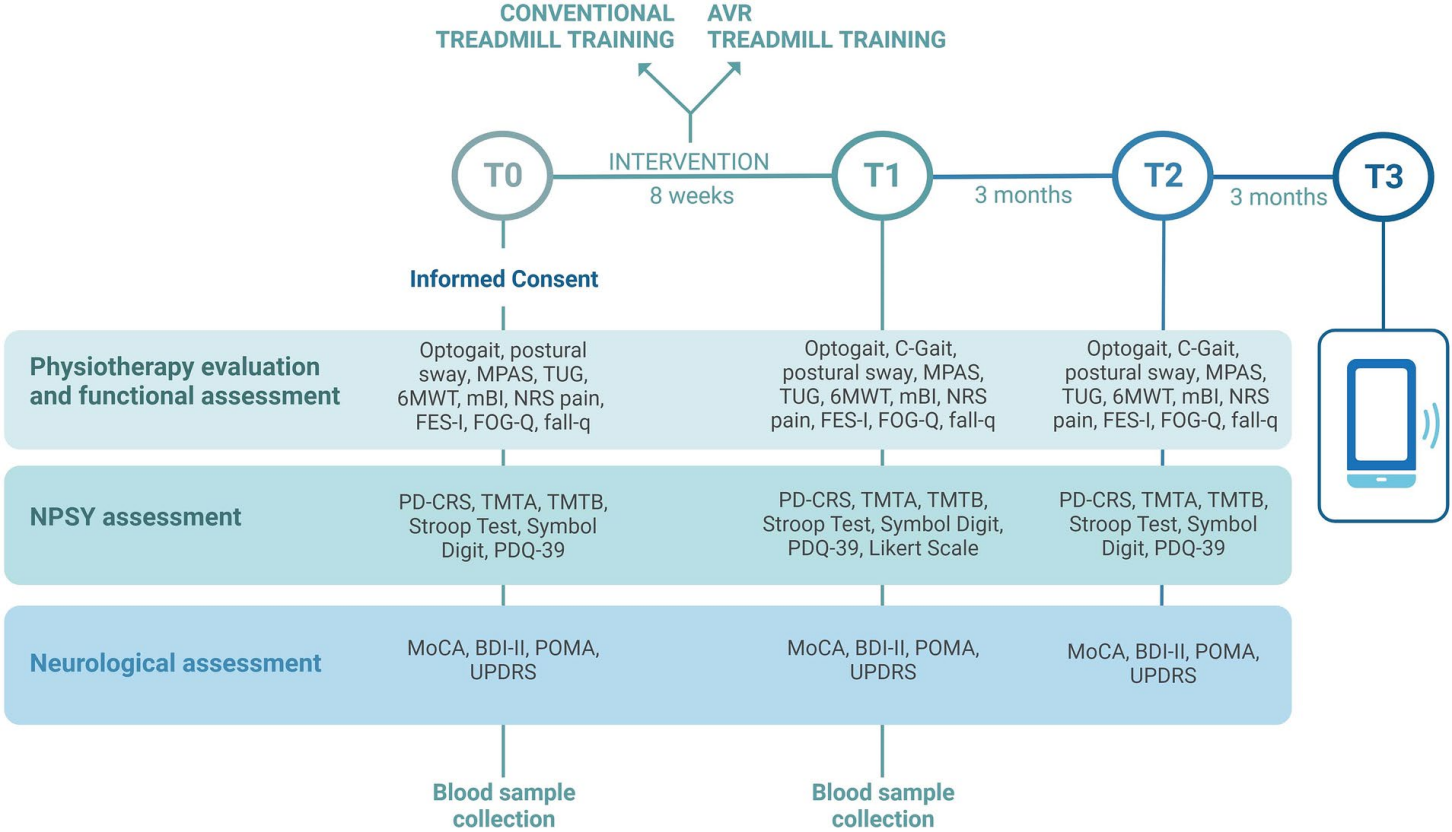
Timing of clinical, instrumental, and biological assessments that will be performed in the VIRTREAD-PD study. Clinical, neuropsychological, and instrumental variables will be collected at baseline (T0), at the end of the treatment (T1), and 3 months after the end of treatment (T2). At 6 months after the end of treatment (T3) a phone interview will be performed. Image created with BioRender.com.

The study is comprehensive of clinical, instrumental, and biological assessments that will be performed by blind raters, in the “on” medication status of patients, at the same time of day for each subject. Assessment timing is reported in Figure 1. Clinical, neuropsychological, and instrumental variables such as postural sway and gait analysis will be collected at baseline (T0), at the end of the treatment (T1), and 3 months after the end of treatment (T2). At 6 months after the end of treatment (T3) a phone interview will be performed to verify the occurrence of falls in a relevant time frame. The same assessment protocol will be performed at each time point (T0, T1, T2) with some exceptions: automatic acquisition of gait parameters using C-Mill in C-Gait mode will be collected only at T1 and T2 in a subgroup of cases; at T1, a 5-point Likert scale will also be administered to register patient satisfaction; during the phone interview at T3 only the falls questionnaire will be administered; biological samples will be collected at T0 and T1.

#### 2.4.1 Clinical assessment

Clinical assessment will be performed by raters experienced in PD patients’ evaluation. The Montreal Cognitive Assessment (MoCA) (40) and Beck Depression Inventory II, Italian version (BDI) (41) will be used to exclude patients with dementia and severe depression. All enrolled participants will be assessed by the following scales/tests:

1. the Performance Oriented Mobility Assessment (POMA) (36) to evaluate gait (POMA-G) and balance (POMA-B)
2. the MDS-UPDRS part III (42, 43) to evaluate different symptoms and signs in PD
3. the Italian version of the Parkinson’s disease cognitive rating scale (PD-CRS) (44) to evaluate global cognitive functions
4. the Trail-Making-Test A and B (TMT-A and B) (45), the Stroop Test (46), and the Symbol Digit Test (SDT) Italian oral version (47) to evaluate attention and executive functions
5. the Parkinson’s Disease Questionnaire (PDQ-39-IT) (48) to evaluate the perceived health-related quality of life
6. the Modified Parkinson Activity Scale (MPAS) (49) and the TUG test (50) to evaluate functional mobility
7. the 6-Minute Walking Test (6MWT) (51) to evaluate walking endurance; the test will be performed indoors along a 30 m corridor, following the American Thoracic Society guidelines (52)
8. the Freezing Of Gait Questionnaire (FOG-Q) (53) to evaluate the frequency and duration of freezing episodes
9. the Falls Efficacy Scale, Italian version (FES-I) (54) to evaluate the fear of falls in different situations of risk
10. the fall questionnaire (55) to account for the number of falls
11. the Numerical Rating Scale (NRS 0–10) (56) to evaluate current pain
12. the modified Barthel Index (mBI) (57) to evaluate functional independence in the basic activities of daily living

At T1, participants will be also asked to rate their satisfaction with the treatment using a 5 Point Likert Scale (range: 1–5, where 5 indicates a very high appreciation).

To avoid inter-rater variability, participants will be evaluated by the same rater (1–2: Neurologist or Physiatrist; 3–5: Neuropsychologist; 6–12: Physiotherapist) at different time points. All scales will be repeated in the same version, except for MoCA, for whom parallel Italian versions are available (58).

#### 2.4.2 Instrumental assessment

Instrument-assisted testing will be aimed at assessing postural sway and overground and treadmill gait. Blinded Physiotherapists will carry out an assessment through a force platform (AMTI OPT464508HF sampling at 1,000 Hz; AMTI, USA), the C-Mill, the Optogait and Witty systems (by Microgate, Italy).^1^

##### 2.4.2.1 Postural sway

The postural sway will be assessed by asking the patient to maintain standing statics with feet together while standing as still as possible in 6 different conditions (feet apart, feet together, feet in tandem position, both with eyes open and eyes closed) lasting 40 s. The assessment will be performed on the force platform and then again on C-Mill.

##### 2.4.2.2 Overground gait

**Figure 2:**
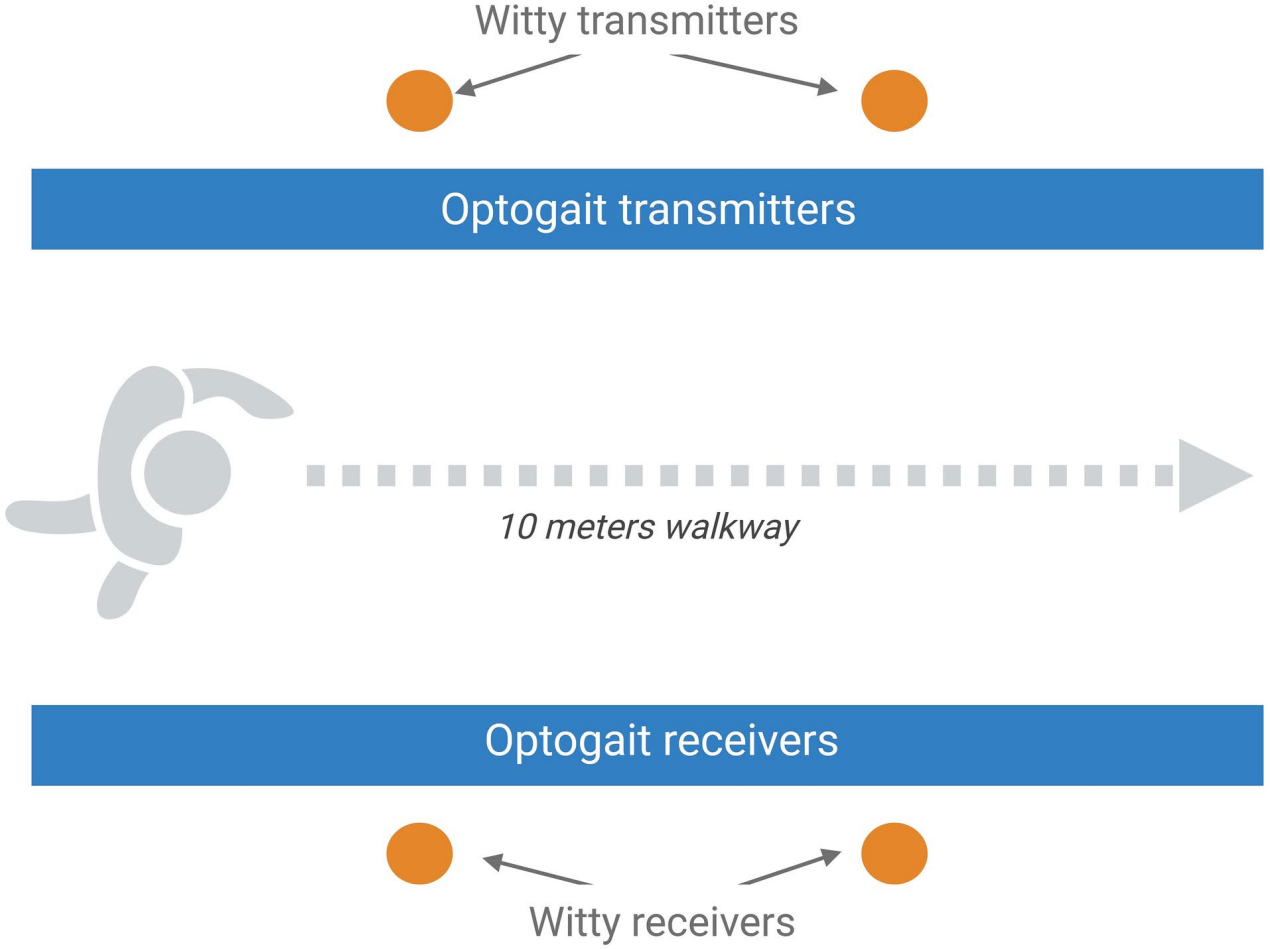
Schematic representation of the setting planned for the overground gait assessment that includes the optical sensing system Optogait and a 10-m Optogait corridor with two pairs of photocells placed to demarcate the central 4 m of the corridor. Image created with BioRender.com.

The overground gait parameters will be evaluated using the Optogait system (Optogait, Microgate S.r.l., Bolzano, Italy) and two pairs of photocells (WITTY, Microgate Srl; Bolzano, Italy; 0.001-s accuracy), as reported in Figure 2. Optogait is an optical sensing system for kinematic gait analysis, consisting of a transmitter bar and a receiver bar. Between the two bars, there is a continuous light beam that is interrupted when the patient’s feet are interposed during gait. The system detects the dimensions of the patient’s foot and estimates the spatial/temporal gait parameters. Witty is a system composed of pairs of photocells that record elapsed split times when the subject crosses the line between the photocells. For the present study, we will use a 10-meter Optogait corridor with two pairs of photocells placed to demarcate the central 4 m of the corridor. Participants will be asked to walk through the corridor at self-selected walking speed (SSWS) and at high speed (HS, defined as the maximal speed that can be safely maintained for a short distance) (51). Three trials at each speed will be performed, with a brief rest between trials, and the mean values will be used for the statistical analysis. All variables will be measured from data recorded in the central 4 m of the corridor, to exclude the acceleration and deceleration periods at the start and end of walking phases, respectively, and thus measure representative steady-state walking data. The following gait parameters will be collected: mean speed, step and stride lengths, cadence and duration of stance, swing, and double-support phases.

##### 2.4.2.3 Treadmill gait

At T1 and T2, all participants will perform the C-Gait test, a preset test of the C-Mill that includes 6 applications of AVR (stepping stones, obstacle mode, walking area, tandem, slalom, and tracks) interspersed with free walking phases. Some applications will be among those exercised by the experimental group during the training and others will be new to participants in this group as well. The C-Gait report provides indications regarding the kinematics during different AVR applications and the success rate of the test.

#### 2.4.3 Biological procedures

Biological samples will be collected at T0 and T1 using serum separation tubes. Forty-five minutes after the blood draw, samples will be separated using centrifugation at 2,500 × *g* for 10 min. Then serum will be aliquoted in cryogen micro vials and stored at −20°C until transfer in dry ice to the LABION, where the analysis will take place.

Serum samples will be used to quantify NfL using the automated immunoassay platform Ella (ProteinSimple, Bio-Techne, MN, USA). Human NF-L Simple Plex assay will be used following the manufacturers’ instructions. A single well will be used for each sample as triplicates assays are automatically performed in Simple Plex assay microfluidic platform.

For cEV isolation, after thawing, serum will be centrifuged at 10,000 × *g* for 10 min. Then, cEVs will be isolated by size exclusion chromatography (SEC) using commercial columns (qEV single Gen2, 70 nm, Izon Science Limited, Christchurch, New Zealand) and Automatic Fraction Collector (AFC, Izon Science Limited). Following the manufacturer’s instructions, 150 μl of serum will be loaded onto the SEC column and cEVs containing fractions will be retained after elution. cEV suspension will be then concentrated by ultracentrifugation (100,000 × *g* for 70 min; L7-65; Rotor SW60; Beckman Coulter, Brea, CA, USA) at 4°C. cEVs will be characterized in agreement with ISEV guidelines (54) by nanoparticle tracking analysis (NTA; NanoSight NS300, Malvern Panalytical, Malvern, UK) and western blot analysis.

Immunoblotting will be performed to characterize cEVs. Briefly, cEV concentrated suspensions from T0 and T1 samples will be obtained by pooling together specimens from multiple subjects. Lysis of cEVs will be performed by sonication, then proteins re-suspended in SDS sample buffer protease inhibitors will be loaded on the electrophoresis gel under reducing conditions. Proteins will be transferred to nitrocellulose membrane and antigens will be probed with primary antibodies specific for cEV protein markers followed by incubation with secondary antibodies conjugated with HRP.

For the Raman analysis, a previously optimized protocol will be followed (34). In brief, cEVs from T0 and T1 samples will be analyzed by means of a Raman micro-spectroscope (LabRAM Aramis, Horiba Jobin Yvon S.A.S, Lille, France). 5 μL of cEVs concentrated suspension will be lied onto a calcium fluoride slide (Crystran, Poole, UK). Spectra will be acquired on the air-dried drop with a 532 nm laser line in the spectral ranges 600–1,800 cm^−1^ and 2,600–3,200 cm^−1^, with the following acquisition settings: 50× objective, 1,800 grooves/mm diffraction grating, 400 μm entrance slit, confocal mode (600 μm pinhole). Instrument calibration will be performed using Silicon reference peak at 520.7 cm^−1^. Labspec6 and Origin2023b (OriginLab Corporation, Northampton, MA, USA) will be used for Raman spectra acquisition, post-processing, and data analysis.

**Figure 3:**
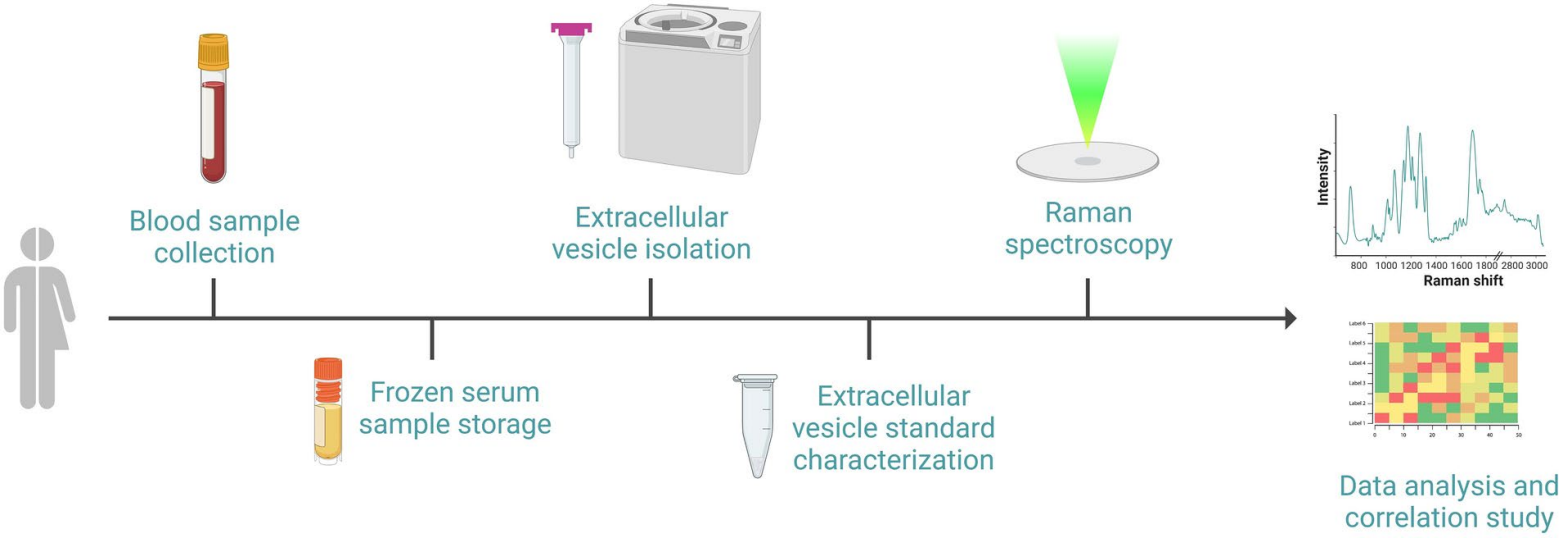
Schematic representation of the sequential events required for the biological procedures: after the recruitment of subjects, blood withdrawal and serum separation will be performed. Serum will be frozen and stored until subsequent cEVs isolation by size exclusion chromatography and ultracentrifugation. cEVs successful isolation will be verified with standard procedure prior to biochemical characterization by Raman spectroscopy using a calcium fluoride substrate and green laser line. Raman spectra will be analyzed and correlated with clinical data. Image created with BioRender.com.

Biological procedures referred to cEV analysis are shown in Figure 3.

### 2.5 Interventional protocol

Intervention in both groups will be performed using the Motek’s C-Mill, with three sessions of treadmill training per week, every day at the same time during the “on” period of the participant, for 8 weeks. Participants will be encouraged to avoid using the side support bars during training, but a brief period (15-min) of familiarization with the task, during which intermittent support on the bars will be allowed, will be conducted during the first three sessions before starting the training. For all subjects, the training will be customized and progressive in gait speed and duration; for the experimental group, the difficulty of AVR applications will also gradually increase. The progression will be based on the participant’s level of performance, but will follow fixed predetermined rules. In detail, gait speed will be set at 80% of the individual’s overground walking speed at the beginning of training, and will be weekly increased by 10% to a maximum of 120% of that speed; at the beginning of training, the training duration will be 25 min, including 5 slots of exercise lasting 5 min, with a 4-min rest between each slot; every 2 weeks it will be increased by 1 min per slot, until a maximal duration of 45 min in the last 2 weeks of treatment. However, the progression will be applied considering the subject’s tolerance, i.e., the ability to keep the heart rate within safety limits and without subjective discomfort, and to walk without having to brace on the side bars.

Participants assigned to the experimental group will exercise five AVR applications (one for each slot of training): “nature island,” “stepping stones,” “walking area,” “obstacles avoidance,” and “tracks.” These applications train balance and changes in walking speed, promote gait adaptation strategies and strategies to overcome freezing of gait, in a safe and controlled environment; moreover, information to promote proper walking is provided, including feedback on gait parameters such as stride length, cadence and symmetry. Five difficulty levels will be prepared for each C-Mill application, with transition between levels set at a success rate of 80%.

In case of discontinuation of treatment (one or more sessions missed), the treatment will be restarted with the gait speed, trial duration, and, for the experimental group, level of difficulty used in the last completed session. During treatment, the participants’ heart rate (HR) will be measured with a chest-band device; in case the heart rate exceeds the safety threshold (75% of HR max, calculated as 220-age for males and 200-age for females), the treadmill speed will be lowered until parameter normalization.

Pharmacological therapy is requested to be stable until the end of the intervention to minimize the effect of external factors on motor, functional, cognitive performances, and biological measures.

### 2.6 Outcome measures

The primary outcome is the difference in gait performance assessed by the POMA-G scale between the TT+AVR and the TT group at T1. The secondary outcomes are differences in mobility (measured by POMA-G at T2 and POMA-B at T1 and T2), in kinematic gait parameters assessed at T1 and T2, measured by the Optogait system, differences in other motor and non-motor symptoms at T1and T2, patients’ satisfaction, assessed with a 5-point Likert scale, and adherence to the treatment.

Exploratory outcome measures include changes in NfL concentration and change in individual Raman spectra of blood-derived cEVs before and after treatment. The correlation between Raman spectra of blood cEVs and the rehabilitation outcome will be assessed (the biological characterization of patients at T0 analyzing cEVs performed by Raman Spectroscopy will provide numerical scores that will be correlated with motor parameters obtained after treatment to identify prognostic biomarkers of rehabilitation outcome).

### 2.7 Data collection and data analysis

#### 2.7.1 Data collection

All clinical data will be collected in the clinical report form (paper CRF). These data will be entered anonymously into a computerized database using REDCap (Research Electronic Data Capture) (59), a web application for building and managing research databases. The use of a dedicated tool is suitable for robust data collection, data quality checks, and limiting missing data. All the users will have private access to the database with personal credentials and dedicated roles. Each patient will be associated with a reference ID and the correspondence between the patient’s name and the associated ID will be stored in a secure external file, to which only clinicians involved in patients’ assessment will have access.

Instrumental data on postural sway and gait analysis will be stored in a computer with restricted access for the personnel dedicated to the analysis of these data. Gait parameters collected using C-Mill in C-Gait mode will be stored in the computer connected to the C-Mill system, which also has restricted access to the staff involved. Biological samples collected at IRCCS Don Carlo Gnocchi in Florence will be sent for analysis, after anonymization, to the LABION at IRCCS Fondazione Don Carlo Gnocchi in Milan.

#### 2.7.2 Data analysis

Statistical analysis will be conducted using the software SPSS v28 (Armonk, NY: IBM Corp). The distribution will be assessed using the Kolmogorov–Smirnov test for all clinical and instrumental continuous data, assuming the presence of normal distribution when *p* > 0.05. Data will then be summarized as mean and standard deviation, median and interquartile range, or absolute and percentage frequency, as appropriate.

The experimental and the control groups will be compared at baseline to explore significant differences in demographic, clinical, and instrumental variables. Parametric or non-parametric tests will be used for continuous variables, according to data distribution. The chi-square test will be used to compare distributions of binary/categorical variables.

Both within and between-group analyses will be conducted to assess at each time point the effects of the treatments delivered, for primary and secondary outcome measures. Specifically, a two-way repeated measures ANOVA will be used with a within-factor (time of assessment) and a between-factor (group).

Regarding biological data, the acquired Raman spectra will be analyzed by multivariate Principal Component Analysis – Linear Discriminant Analysis (PCA-LDA) classification. PCA will reduce the number of variables into principal components, which will be used to build the LDA model. The model will be built to discriminate clinical improvement detected as quantifiable change on clinical scales, defined based on the minimum clinically important difference, when available from the literature. The sensitivity, specificity, and accuracy of the predicting model based on spectra data will be assessed. In addition, correlation analysis will be conducted to evaluate the association between spectral data and change on clinical scales recorded between the treatment’s beginning and end. Statistical analysis of the Raman data will be performed using Origin2023b (OriginLab Corporation, Northampton, MA, USA) (34).

Univariate analysis models will be used to select the best prognostic markers of clinical improvement to be included in a multivariate model.

For all analyses, statistical significance will be set at *p* < 0.05.

## 3 Discussion

In this study, we propose to compare the effects of TT+AVR and TT, delivered according to a customized progressive rehabilitation protocol, on gait and other motor and non-motor symptoms in patients with II-III stage PD and gaits disturbance, testing NfL and cEVs as biomarkers of neuroplasticity and prognostic biomarkers, respectively.

As to the definition of the primary outcome, we searched for a validated and recommended clinical measure of gait that could be easily collectable in all clinical settings, and, following the Parkinson EDGE Task Force Recommendations (60, 61) and according to the literature (62, 63), we chose to use the POMA as the primary measure to assess gait and balance. POMA scale has the advantage of testing both gait and balance providing separate scores for each variable of interest.

However,. although largely used, this scale estimate only basic gait parameters (i.e., speed), not providing information on the pattern or quality of the movement. Also, it has been reported to have low sensitivity, and to be influenced by instruction and tester bias (62). For these considerations, in order to capture the gist of individual gait changes associated with disease progression or with different rehabilitation programs, we decided to include a series of kinematic parameters, detectable using Optogait, as also recommended for research in PD rehabilitation (3). Indeed, pooled data from a recent metanalysis show a moderately large effect on gait speed in favor of TT when compared with no exercise or sham treatment in PD (9). This result has been replicated in a more recent systematic review with network meta-analysis, according to which TT resulted more effective compared to control in improving gait speed and walking distance, highlighting that the body weight support-TT is superior compared to control and many exercise types in improving gait speed, walking distance, balance and motor symptoms (64). In this regard, gait speed, although not specific to PD assessment (3), has been described as “almost the perfect measure” (65) since it is a reliable and sensitive measure that correlates with functional ability and balance confidence. This evidence suggests the importance of including measures of walking speed in the motor assessment together with other spatial–temporal parameters, such as cadence and step length, to correctly interpret the quality of gait. The same considerations apply for the assessment of balance by the POMA-B, this is why we have introduced the postural sway assessment by using the force platform.

A recent systematic review (66) found a very high variability in the distance adopted to measure gait parameters in PD, that ranged from 1.4 to 100 m, with about 22% of studies that used short (up to 6 m) distances. In the present study, the choice of a 4 m distance is also due to technical constraints. We will use a 10-m Optogait corridor, in which two cross bars need to be placed at the beginning and end of the corridor; therefore, the subject should start walking within the corridor and stop before passing the final bar. To measure parameters during steady-state walking, the data from the initial 3 m (acceleration) and final 3 m (deceleration) must be eliminated, and only the middle 4 m are available for measurement. However, we will record three trials for each speed, taking the average for all analyses. Both features (recording the steady-state walk and averaging between three trials) should yield representative measures of the subject’s walk.

In addition to measuring gait parameters along a short distance, we also chose the 6MWT as a test to assess aerobic capacity/endurance. This test has been validated in PD (51).

Still referring to the walking and balance assessment, attention must be paid to the C-Gait. This innovative test allows for recording data during different AVR applications to detect specific gait dysfunction. The C-Gait allows to distinguish between people with FOG and those without, outperforming traditional walking tests, being obstacle avoidance and speed of adaptation items suggestive of FOG with high sensitivity (67). C-Gait assessment will be performed at T1 e T2 in both the control and experimental groups to assess whether performances on gait adaptability are superior in the experimental group and whether this gap is maintained at 3-month follow-up.

As to the other clinical measures, we chose the MDS-UPDRS part III to assess motor symptoms severity. Then, we selected a set of questionnaires and performance tests according to the “European Physiotherapy Guideline for Parkinson’s Disease” recommendations (68). Since the importance of tracking falls in PD due to their frequency and consequences on everyday activities, fear of falling, and QoL (69–72), we included the fall questionnaire, ensuring to report the number of falls, modality, and related consequences, together with the FES-I questionnaire, to evaluate fear of falling. The incident rate of falls over 6 months after the end of treatment will be compared with those referred to the 6 months before the rehabilitation treatment.

In the same way, FOG is a common (63% of PD patients have FOG) and complex gait disorder in PD that frequently causes falls and falls-related anxiety, both of which lower QoL and functional independence, cause shame and dissatisfaction promoting social stigmatization and disability (73–75). Thus, in order to assess this symptom, we included in the tests’ battery the FOG-Q, the only validated tool available to subjectively assess the severity and frequency of FOG (76).

To assess the level of functional mobility the TUG and the MPAS have been selected according to the literature (77). The TUG has the advantage of testing three anchors of functional mobility (gait, balance, and transfers), and provides also indirect estimation of the risk of falls; however, it assesses the patient’s capacity in a neutral setting, rather than performance in daily living activities. For this reason, we adopted the MPAS, a scale specifically designed to evaluate functional gait, balance and transfers through different scenarios in daily activities. The major limitation of MPAS is the number of accessories, space, and time needed to perform the test which may not be practical or feasible in all centers.

Pain is a frequent non-motor symptom in PD, contributing significantly to disability and reduced QoL. It affects around 67.6% of PD patients versus 15–30% of the general population. The most frequent pain syndromes in PD are musculoskeletal pain, neuropathic radicular pain, dystonia-related pain, akathitic discomfort, and primary central parkinsonian pain (78). Among clinical scales suitable to assess pain intensity, the NRS scale is simple, well-validated, and easy to use for clinicians and to understand for patients. The NRS is a recommended tool for assessing pain intensity in the general population and is widely used also in PD, although it is cautiously recommended in this population because it is not disease-specific (78).

Experience appreciation will be estimated for both treatment groups using a 5-point Likert scale and correlated with the level of adherence to the treatment. Indeed, independently of the effectiveness on motor symptoms, results coming from previous studies suggest a higher appreciation of VR training by the patients compared to the conventional TT which is perceived as monotonous or boring, and less useful to integrate into their daily life environment (79–81). Thus, satisfaction will be tested to confirm these findings. Along with adherence to either treatment, and to the evaluation of the possibly different clinical and instrumental effect of either intervention, this measure can provide relevant information on potential barriers and motivators for exercise and rehabilitation. Indeed, as to accessibility to these services, while the TT is currently available in most rehabilitation facilities, TT associated to AVR is more expensive and less available. As to motivators, AVR is claimed to be a relevant motivator (14): our study will allow us to investigate this issue, by allowing a comparison of both adherence and patients’ satisfaction in either intervention. Thus, our study may provide highly relevant information about the superiority of TT + AVR versus TT, to potentially provide grounds to expand the adoption of TT + AVR instruments, even if this may be associated to generally higher costs.

According to multidimensional bio-psycho-social assessment and approach recommended by the International Classification of Functioning, Disability and Health (ICF), and the strict interconnection between motor, psychological, cognitive functions and QoL in PD, the present VIRTREAD-PD protocol includes also an extensive psychological and neuropsychological assessment (82–84).

Several studies demonstrated the positive effect of both specific and non-specific physical activity on cognitive functions in PD, especially on executive functions (85–87). Recently Pelosin et al. (79) showed how a longer TT with VR, up to 12 weeks, improves not only motor but also executive functions, visuospatial ability, and attention. On the other side, the baseline cognitive status, specifically memory impairment, seems to influence the response to gait rehabilitation in PD (88). For these reasons, a feasible yet exhaustive clinical neuropsychological assessment is mandatory in the development of a rehabilitation program. In the present study, the MOCA and the PD-CRS were chosen among Level I cognitive assessments proposed by Litvan et al. (89) to identify Mild Cognitive Impairment (MCI) in PD. The PD-CRS was preferred to the Scales for Outcomes in Parkinson’s Disease-COGnition (SCOPA-Cog), because it assesses both frontal-subcortical functions (i.e., sustained attention, working memory, alternating and action verbal fluencies, clock drawing, and immediate and delayed free-recall verbal memory) and instrumental-cortical functions (i.e., confrontation naming, copying a clock). PD-CRS further overcomes the limitations of Level II neuropsychological assessment, not always applicable in clinical settings for PD patients who are more likely to have attentive lability and fatigue (44). PD-CRS ultimately allows to discriminate between cognitively intact PD patients and PD patients with MCI or dementia, and between the latter (90).

Moreover, since not only motor but also cognitive differences in PD subtypes may influence motor performances and PD rehabilitation outcomes (91), this prompted researchers to advocate for cognitive testing as an essential component of the VIRTREAD-PD protocol, to investigate whether cognitive features at baseline may influence the trajectory of recovery across the study’s timepoints.

The effect of physical activity on depression and QoL in healthy aging and in PD has been widely documented; in detail, aerobic training exercise significantly improved the scores not only on the MDS-UPDRS, but also on the BDI, and the QoL scales (83). Accordingly, in the present protocol, we included measures of depression and of QoL, to explore whether TT+AVR could affect also these psychological variables. The described multidimensional approach will allow for an in-depth study of the aspects inherent in the interaction between the motor and cognitive spheres. Indeed, rehabilitation is an active procedure that at a cellular level involves neuroplasticity mechanisms (8) and that at an individual level requires the ability to follow, understand, and recall precise instructions involving motor learning processes.

Indeed, due to experience-dependent neuroplasticity, exercises that combine aerobic activity with goal-based training have the potential to enhance both the cognitive and automatic components of motor control in people with mild to moderate stages of the disease (92). Moreover, innovative training targeting cognitive components of complex motor actions (ie multitask conditions) enables functional gait improvement (23). The process by which this kind of training improves walking ability is mainly guided by the activation of compensatory cortical mechanisms such as increased attention and use of visual cues, linked to motor learning processes (93). Since in similar previous randomized clinical trials biological data has not been collected or not analyzed with the intent to find biomarkers of neurorehabilitation, a major innovation of VIRTREAD-PD protocol is the inclusion of NfL quantification and cEVs analysis through Raman spectroscopy. NfL are now attracting considerable attention as biomarkers of axonal damage and neurodegeneration, as well as biomarkers of therapy effectiveness that could represent an objective complement to other process of measuring clinical outcomes (94) thanks to the availability of robust automatable assays for their quantification (95, 96). On the contrary, the analysis of cEVs represents a major innovation in the present protocol. Indeed, cEVs include multiple subpopulations of EVs released by all body cells, including those released by neurons and glial cells (97). It was already demonstrated their major involvement in the reparative mechanisms occurring in the brain after a lesion (98, 99), but the complex composition of cEVs, their heterogeneity, their nanoscale dimensions and the laborious isolation and characterization methods have currently limited their application in a clinical scenario. Nonetheless, Raman spectroscopy can represent a turning point in the use of cEVs in clinics, allowing to condense in a continuous variable, information coming from multiple molecular compounds. This method has been already utilized in PD for diagnostic purposes, revealing abnormalities in the plasma spectroscopic fingerprint compared to healthy controls (100) as well as in the discrimination of people with PD from control subjects by means of cEV Raman fingerprint (34). Although PD may be diagnosed clinically with a high degree of confidence, predicting its progression rate remains difficult since the condition is clinically heterogeneous and an objective marker indicative of cumulative disability and of the recovery following treatment is currently lacking. Biological signatures obtained utilizing this method for patient stratification before and after treatment could solve this currently unmet need. Indeed, rehabilitation recovery involves a plethora of complex biological mechanisms that include neuroplasticity, brain remodeling, and brain-muscle communication (101) that can be collectively monitored by exploiting the Raman cEV profile.

Concerning treatment, the main innovation in VIRTREAD-PD is the use of the TT+AVR. As in Mirelman (24), we structured an incremental protocol for the time and duration of 5 walking slots in the rehabilitation sessions. In order to administer treatments tailored to the performance of the individuals involved in the study, training progression was planned in line with a predefined progression plan. Due to the intimate association between motor performances and attentive/executive functioning in PD and as suggested by previous research (23), we included the “obstacle avoidance” task among the AVR applications, targeting the “motor-cognitive interactions,” to improve gait performances and reduce fall risk. Duration and frequency of training are planned as reported in the literature as effective on gait and balance (ranging from 4 to 8 weeks, including 12–20 sessions) (11, 17). In conclusion, this study will compare the effects of 2 rehabilitation protocols on motor symptoms in PD. To our knowledge, this is the first randomized controlled study specifically designed to enroll only PD patients where the effect of conventional TT is compared with that of TT with AVR applications. Innovative elements and strength points of the protocol are represented by a multidimensional approach using both clinical and instrumental assessments, the predefinition of an algorithm for treatment customization, and the application of an innovative method to explore potential biomarkers of neuroplasticity and potential prognostic biomarkers. The results of this study will provide new insight to personalize and possibly optimize the effects of technological rehabilitation for PD patients.

## Ethics statement

The study was approved by the “Comitato Etico Regione Toscana – Area Vasta Centro”. The study will be conducted in accordance with the local legislation and institutional requirements. The participants will provide their written informed consent to participate in this study.

## Author contributions

GL: Conceptualization, Investigation, Methodology, Supervision, Writing – original draft, Writing – review & editing. MBa: Conceptualization, Resources, Supervision, Writing – review & editing. AG: Conceptualization, Data curation, Investigation, Project administration, Writing – original draft. SPa: Data curation, Formal analysis, Methodology, Writing – review & editing. SC: Data curation, Software, Supervision, Writing – original draft. SD: Conceptualization, Investigation, Project administration, Writing – original draft. DL: Data curation, Investigation, Writing – review & editing. AM: Conceptualization, Methodology, Writing – original draft. GC: Investigation, Methodology, Supervision, Writing – original draft. MP: Data curation, Investigation, Project administration, Writing – original draft. TC: Conceptualization, Investigation, Project administration, Supervision, Writing – original draft. CP: Investigation, Supervision, Writing – original draft. SPi: Data curation, Investigation, Writing – original draft. CS: Data curation, Investigation, Writing – original draft. MBe: Conceptualization, Data curation, Methodology, Supervision, Writing – review & editing. CM: Supervision, Writing – review & editing. SR: Conceptualization, Methodology, Supervision, Writing – review & editing. FC: Conceptualization, Funding acquisition, Project administration, Resources, Supervision, Writing – review & editing.

## Footnotes

^1^ http://www.optogait.com/Gait-Phases
